# Non-surgical organ preservation and new technologies in laryngeal radiation

**DOI:** 10.3389/fonc.2024.1494854

**Published:** 2025-03-17

**Authors:** Andreas Dietz, Hans Eckel, Alexander deVries, Markus Brunner

**Affiliations:** ^1^ Hals-Nasen-Ohren-Universitätsklinik, Leipzig, Germany; ^2^ Hals-Nasen-Ohrenklinik, KABEK Klinikum Klagenfurt, Klagenfurt, Austria; ^3^ Klinik für Radioonkologie und Strahlentherapie, Vorarlberger Landeskrankenhäuser Feldkirch, Feldkirch, Austria; ^4^ Klinische Abteilung für Hals-, Nasen-, Ohrenkrankheiten, Universitätsklinikum Krems, Krems, Austria

**Keywords:** larynx organ preservation, induction chemotherapy, laryngectomy, head and neck cancer, non-surgical treatment

## Abstract

The term “larynx organ preservation” (LOP) has become a synonym for non-surgical laryngeal cancer treatment based on chemotherapy and radiation multimodality therapy [simultaneous chemoradiation (CRT) or neoadjuvant chemotherapy followed by radiotherapy (NCT+RT)]. Currently, the distinction between good and bad candidates for LOP is not clear, and the decision for surgical or non-surgical treatment depends on the patient’s needs and desires, the experience and recommendation of the surgeon, the philosophy of the institution, and others. Nevertheless, the major disadvantage of LOP by CRT and NCT+RT is the potential need for salvage surgery due to tumor persistence after the application of full per-protocol treatment. Head and neck surgeons worldwide complain that in principle, salvage surgery is frequently possible after CRT but causes major complications and is not feasible in a relevant number of patients. While NCT+RT is globally used to select responders for LOP, NCT alone has not been shown to improve overall survival. Therefore, this procedure has lost its influence in standard head and neck cancer treatment beyond LOP. Recently, NCT as part of the perioperative transoral surgical treatment concept in head and neck cancer is gaining interest again. In addition to conventional chemotherapy, the combination with immune checkpoint inhibitors as a neoadjuvant concept has shown to be effective in non-controlled trials by opening a new door of encouraging treatment options for LOP.

## 1 Introduction

The term “larynx organ preservation” (LOP) has become a synonym for non-surgical treatment of laryngeal cancer based on chemotherapy and radiation multimodality therapy. Beyond doubt, larynx-preserving surgery for laryngeal and some hypopharyngeal cancers, which covers a wide spectrum of technical options [transoral laser microsurgery (TLM); open procedures] up to some T4 stages for highly experienced surgeons, is very worthwhile (1, 2). In the locoregional advanced group of laryngeal and hypopharyngeal head and neck squamous cell carcinoma (LHSCC), there are two kinds of patients: those who are candidates for functional larynx organ preservation by avoiding ablative surgery and those who are not. Currently, the distinction between them is not clear, and the decision depends on the patient’s needs and desires, the experience and recommendation of the surgeon, the philosophy of the institution, and others. Nevertheless, the interdisciplinary debate concerning this issue is partly polarizing: some authors suggested that increasing non-surgical treatment of laryngeal cancer was responsible for decreasing the survival of these patients in the last two decades in the USA (3).

Neoadjuvant chemotherapy (NCT) followed by radiotherapy was predictive for outcomes after radiotherapy and also led to long-term cure rates and offered NCT in larynx preservation concepts in the late 1970s (4). Moreover, this observation led to the conception of two large randomized studies in the 1980s, which compared induction chemotherapy followed by radiotherapy with a primary total laryngectomy and postoperative radiotherapy (5, 6). Meta-analysis suggested that a concomitant randomized controlled trial (RCT) is more effective than a sequential RCT (7). This led to the conduction of the RTOG 91-11 trial, a large, randomized, three-arm multicenter study comparing NCT followed by radiotherapy (RT) with concomitant RCT or RT alone (8). These results recommended simultaneous chemoradiation as the optimal concept (until today) based on level 1 evidence. Over the years, observations of late side effects like severe dysphagia, tracheotomy requiring larynx edema, and increasing postoperative complications following salvage surgery led to uncertainty and rejection of these aggressive organ-sparing protocols in many surgery-driven centers worldwide (1).

Addressing these important problems, third-generation LOP protocols are promoting again induction chemotherapy following radiation alone to avoid concomitant spilling of function-limiting late toxicities due to simultaneous chemoradiation (9). Taxane-containing regimens were shown to be more effective compared to “older” platin-based induction chemo regimens (10). Nevertheless, NCT in head and neck cancer was replaced mainly by definitive radiochemotherapy or upfront surgery with or without adjuvant treatment in advanced diseases due to the lack of survival benefits in the last two decades (11). Recently, the additional use of immune checkpoint inhibitors in neoadjuvant concepts [neoadjuvant immune chemotherapy (NICT)] with sub-sequential surgery in clinical trials stimulates several questions addressing the standard of resection margins after induction and handling of different degrees of surgical aggressiveness depending on the level of response. Furthermore, the specific biological behavior of tumor shrinkage and the definition of the former tumor bed after induction are under critical consideration.

## 2 Current view on larynx organ preservation programs

LOP in locally advanced LHSCC is very desirable, although total laryngectomy represents an effective treatment strategy. Current treatment options to preserve the larynx and its function include primary concurrent chemoradiotherapy or induction chemotherapy followed by radiotherapy. The “VA” trial (5) established induction and subsequent radiation utilizing PF [cisplatin (P) plus 5-fluorouracil (F)] for induction as an appropriate alternative to total laryngectomy and achieved 35% LOP. Despite verification of this finding, e.g., in the EORTC 24891 trial (6) in hypopharynx carcinoma, induction chemotherapy and subsequent radiotherapy have not been accepted since cisplatin-based simultaneous chemoradiation (CRT) is recommended for LOP based on the findings of the RTOG 91-11 trial (8, 12, 13).

In patients who receive optimal combinations of chemotherapy and radiotherapy (NCT or CRT) without violation of the treatment protocol, both total laryngectomy and chemoradiation offer similar outcomes in T3 but not T4a disease (14). Radiotherapy alone should no longer be considered an option for these tumors (15, 16).

Since RTOG 91-11 lacks current state-of-the-art functional follow-up screening regarding dysphagia, voice, and any late toxicity assessment, the relevant definition of functional organ preservation was not met and was interpreted as only “organ in place”. Keeping all these factors in mind, Lefebvre and Ang (17) defined the still relevant goal for future larynx organ preservation trials as “laryngoesophageal dysfunction-free survival” and limited the indication to big T3s, indicating that T4a may end up with lower functional preservation rates. Grover et al. (18) presented retrospective National Cancer Database data from 969 patients suffering from T4a laryngeal carcinoma (M0, treated between 2003 and 2006) that showed significantly better 5-year overall survival (p = 0.001) in patients receiving primary surgery (36% received TL with or without adjuvant radio/chemotherapy, and 64% received primary chemoradiotherapy). Analysis of T4 laryngeal cancer data from high-volume centers such as the MD Anderson Cancer Center or Netherlands Cancer Institute demonstrated significantly better local recurrent-free survival after TL and unacceptable functional outcomes after CRT in this patient group (19, 20). Patients treated with initial laryngectomy had more distant metastases and no overall survival benefit (p = 0.7).

**Figure 1:**
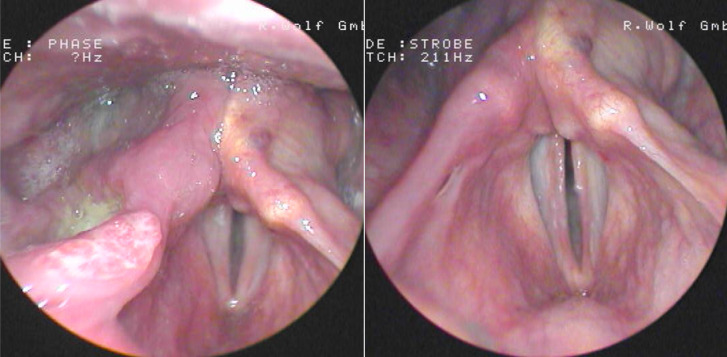
Patient with T4a hypopharynx carcinoma before (left) and after (right) one cycle TPF showing complete remission in early endoscopic response evaluation. Patient was treated according to the DeLOS-II protocol [induction chemotherapy (IC) followed by radiotherapy (RT) versus cetuximab plus IC and RT in advanced laryngeal/hypopharyngeal cancer resectable only by total laryngectomy] (25).

Nevertheless, PF-based induction followed by radiation was superior to CRT in the RTOG 91-11 trial by causing less severe late toxicity and increasing laryngectomy-free survival due to fewer non-cancer deaths (21). Moreover, CRT was associated with increased late toxicity and impaired survival, especially in T4a LHSCC patients (18, 19). The potential need for salvage surgery due to tumor persistence after full per-protocol treatment proved to be a major disadvantage of LOP. Late salvage total laryngectomy after CRT or radiotherapy causes major complications and is often not feasible (22, 23). Therefore, early identification of patients unlikely to benefit from LOP attempts is needed to spare the consequences from complete CRT or radiotherapy plus salvage surgery. Since new multimodal treatment protocols and chemotherapies including targeted therapies have been emerging (24), further development of LOP by induction and radiation has remained under consideration. One important field of interest is early response evaluation as a clinical predictor for positive outcomes, which was demonstrated in the DeLOS-II trial by exploring early response by transoral office endoscopy just after the first cycle of induction with TPF/TP (25; **Figure 1**). Ongoing trials like the PRESERVE study are interesting in this context (26).

TAX 323 (27) revealed induction with TPF, the combination of docetaxel (T) with PF, being superior to PF alone. The GORTEC 2000-01 trial demonstrated the superiority of TPF in LOP (9). The DeLOS-I trial showed the efficacy of TP-based (carboplatin plus paclitaxel) IC + RT with low late dysphagia rates (28). However, the literature is consistent that induction before radiotherapy or CRT prolongs progression-free survival but does not improve overall survival significantly (11). The EHNS-ESMO-ESTRO guidelines recommend with evidence level II grade of recommendation A (II A evidence) exclusively TPF induction followed by radiotherapy in responsive patients as an option for LOP in local advanced LHSCC otherwise requiring TL (29, 30). Several induction trials may have underestimated the potential efficacy of a full TPF regimen. In this context, the ongoing SALTORL trial, which includes only patients able to tolerate a complete TPF treatment cycle, may provide valuable insight for future studies (31).

## 3 Current view on when T3 laryngeal and hypopharyngeal cancers and the important role of pretreatment function: surgical or non-surgical treatment

While stage I–II glottic and supraglottic cancer can be cured with (open or endoscopic) partial laryngectomy or primary radiotherapy, the treatment of stage III–IV laryngeal cancer remains a matter of individual consideration for each patient.

Although advanced partial laryngectomies can successfully be applied to individual cases (32–35), the vast majority of advanced laryngeal and hypopharyngeal cancers will not be considered candidates for these procedures. The reason is that most T3 carcinomas feature fixed vocal cords, indicating a considerable risk for crico-arytenoid joint or even cricoid cartilage infiltration. Such tumor extensions are notoriously difficult to treat with organ-preserving surgical procedures (35). Addressing this point more specifically, the distinction between arytenoid fixation and vocal fold fixation is key. A vocal cord may be fixed without the arytenoid being fixed, which significantly impacts treatment choices and results. Moreover, Ary fixation is a subjective evaluation with high variability and therefore should be evaluated precisely. The evaluation of posterior paraglottic space involvement seems to be a more reproducible surrogate (in this context, we refer to the review of Cesare P. et al. in this collection) (36–39). In addition, there is still major concern relating to postoperative radiotherapy or chemoradiation for fear of laryngeal chondritis and unfavorable functional outcomes after partial laryngectomies (34).

For T3 hypopharyngeal cancer, the EHNS-ESMO-ESTRO guidelines propose, in general, adjustment from laryngeal cancer (low evidence in hypopharyngeal cancer; the only controlled prospective trial was the EORTC NCT trial, 6) concomitant chemoradiation as the standard of care for all patients whose tumors would require total laryngectomy (30). For those not requiring total laryngectomy, conservative (laser) surgery followed by RT or CRT is also mentioned as standard treatment (30). Advanced open partial laryngectomy procedures, such as supracricoid or supratracheal partial laryngectomies, are suitable options for these patients (34). The MD Anderson Cancer Center recommendation was in line with the Lefebvre and Ang recommendation to limit non-surgical indications to T3 and small selected T4a cancers. Additionally, some sound findings were described in a large Netherlands observational study showing that the non-surgical organ preservation approach is only survival equivalent in T3 but not T4a cancers compared to total laryngectomy with 42% vs. 48% after 5 years (20).

The loss of laryngeal function after total laryngectomy has become unacceptable for many patients, and overall survival rates have not consistently been superior to organ-preserving chemoradiation. In fit patients with unimpaired deglutition, combinations of chemotherapy and radiotherapy are consequently now considered the optimal treatment for most T3 laryngeal and hypopharyngeal cancer patients, although an individualized approach to decision making is still essential (40). Furthermore, the impact of nodal burden on treatment selection seems to be underestimated. In modern laryngeal oncology, decision making relying solely on cancer staging may be misleading (41). Apart from patient preferences and locally available expertise regarding different therapeutic approaches, performance status, previous oncological therapies, and relevant comorbidities will lead the way to making adequate decisions. Assessing comorbidities with the TALK score (T-stage, Albumin, Liquor, Karnofsky Performance Status) to include tobacco use during treatment may be a simple yet highly conclusive tool to assess patient characteristics (42–44). For patients with significant comorbidities not qualifying for chemotherapy as a part of their initial treatment, total laryngectomy is still a valid option (45). Additionally, early response evaluation during induction chemotherapy proved to be an effective selector for successful larynx organ preservation (25).

## 4 Surgical view on new resection margins after neoadjuvant treatment in advanced laryngeal and hypopharyngeal cancers since surgery after NCT is still under strong consideration

The principle of en-bloc R0 resection is favored for high-level surgery in head and neck oncology. While en-bloc resection remains a key surgical principle, the surgical extension into more complex anatomical areas and technological advances [i.e., endoscopic mucosal resections, TLM, or transoral robotic surgery (TORS)] highlights the need to reconsider the potential merits of piecemeal tumor removal (46, 47). Piecemeal resections are controversial because they result in fragmentation of the removed specimen, compromising its integrity and complicating confident histopathologic evaluation for the adequacy of excision. While in certain areas, such as the skull base or larynx, piecemeal tumor removal may be justified by anatomical constraints (48), or functional imperatives (49) (i.e., preservation of voice and deglutition), the apparent increasing use of the piecemeal approach in anatomically simpler and more accessible parts of the human body is more difficult to understand (50). Quality initiatives by the American Head and Neck Society (AHNS) emphasize the importance of obtaining a negative margin (R0) in head and neck squamous cell carcinoma (HNSCC) (51). However, the approach to margin sampling varies considerably from surgeon to surgeon (52, 53). Trials addressing surgery of oral cavity HNSCC could demonstrate that reliance on margin sampling from the tumor bed was associated with significantly worse local control, most likely owing to narrower margin clearance and greater incidence of positive margins. A resection specimen-based margin assessment is recommended (50, 54).

Nevertheless, all mentioned observations are based on primary surgery experiences in treatment-naive patients. The situation of surgery after NCT is still under strong consideration. The pathological response to NCT can potentially affect the evaluation of surgical margins, due to a non-centripetal widespread cell dropout throughout the tumor mass, which determines a more challenging assessment of tumor infiltration.

**Figure 2:**
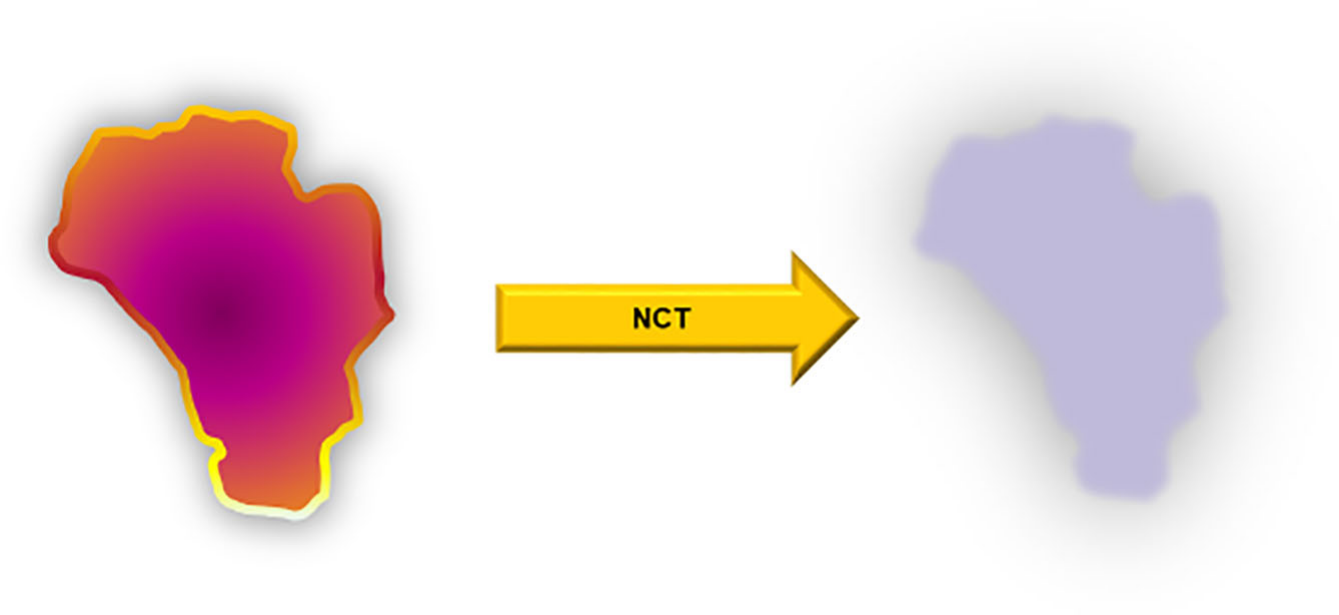
Principle view on a given head and neck squamous cell carcinoma (HNSCC) treated with neoadjuvant chemotherapy (NCT). The ideal result is on the right side as a gray shadow of the vital tumor cell-free tumor bed.

**Figure 3:**
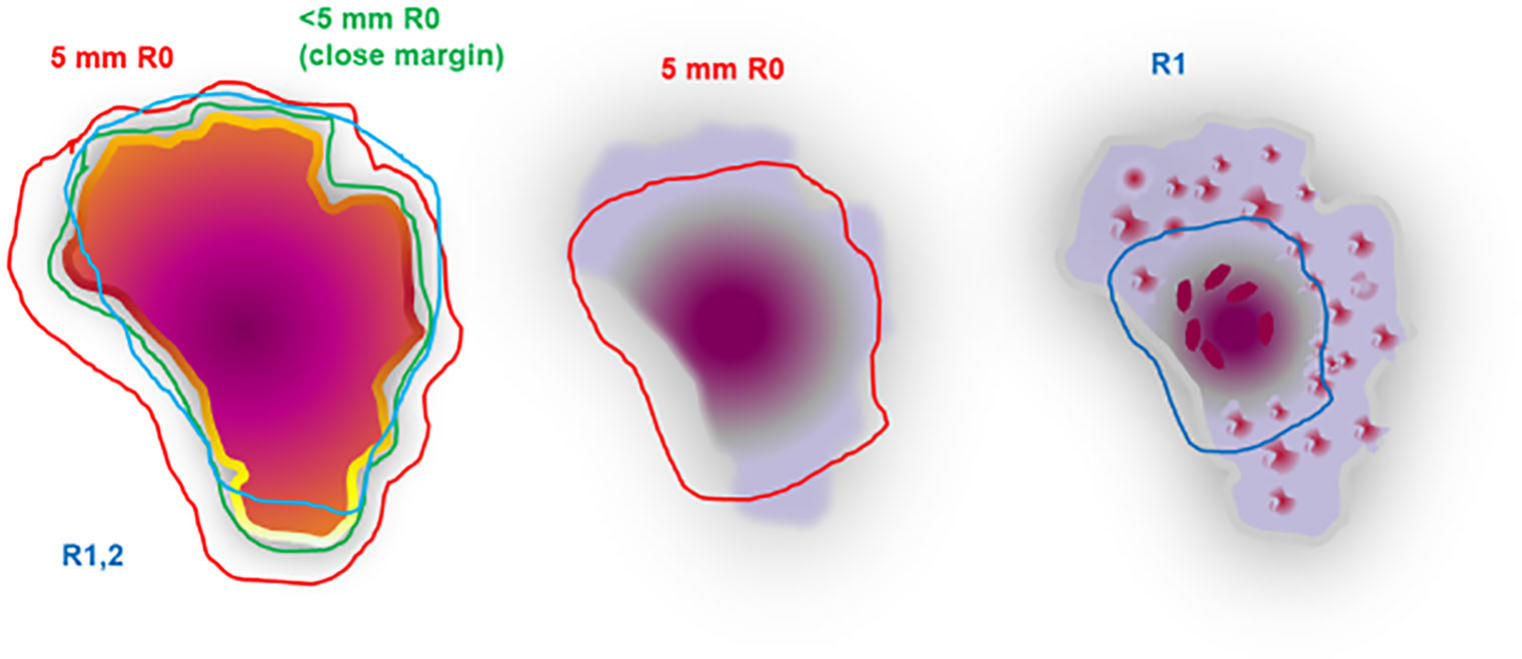
Tumor pattern for classification of the margins. Picture left side: different resection lines to characterize the margins R0 5 mm, R0 < 5 mm, R1, 2. Picture mid side: if the tumor shrinks homogeneously without any remnant tumor islands, a closer resection line would end up correctly in R0 (downstaging). Picture right side: tumor shrinkage with remnant tumor islands; a smaller resection line would result in R1 resection (downstaging with reduced surgical extent in this situation is not acceptable).

Therefore, many surgeons are convinced that any reduction of the original margins after NCT (downstaging) should be avoided because of the high risk of remnant tumor islands in the former tumor bed. The major surgical opinion is still resection in old margins (principle demonstration of tumor pattern after NCT are shown in **Figures 2**, **3**).

## 5 New technologies for radiation of advanced laryngeal and hypopharyngeal cancers

Functional organ preservation with concurrent chemoradiation or induction chemotherapy followed by radiotherapy is the internationally recommended treatment alternative for total laryngectomy. After non-surgical protocols, approximately 30%–40% of these patients will lose functionality of the larynx either by salvage total laryngectomy or by side effects of the treatment. Therefore, the area of interest is how to select the patient for the optimal treatment strategy. Milinis et al. showed that current smoking significantly increases the risk of non-functional larynx, and pretreatment hemi-larynx fixation was found to be associated with a significantly increased risk of locoregional recurrence (55). As mentioned above, TALK score and early response evaluation are helpful in predicting outcomes (25, 42). Here, we present a short choice of selected new radiation strategies (beyond induction chemotherapy) that can help to individualize therapy.

### 5.1 Adaptive radiotherapy

Adaptive RT is the process of re-planning patients during treatment in response to observed spatial and structural changes, e.g., weight loss and changes in tumor volumes. Therefore, adaptive RT allows modifications of the radiation plan based on changes that occur during treatment. An example is volumetric reductions in tumoral volumes, resulting in unintended dosimetric changes affecting the treatment efficacy and overdosing normal organs, which would ultimately result in increased toxicity (56, 57).

### 5.2 MRI-guided radiotherapy

MRI in laryngeal cancer can evaluate tumor extension/invasion into anatomical structures such as the pre- and paraglottic space, cricoarytenoid unit, and subglottic and base of tongue regions especially in locally advanced tumors. In addition, it could be helpful to assess perineural tumor spread and vascular involvement. Therefore, it could be helpful in a more precise, tumor-adapted radiation therapy (58).

### 5.3 Unilateral neck irradiation

Bilateral elective nodal irradiation (ENI) remains the standard treatment for head and neck squamous cell carcinoma. However, diagnostic imaging techniques have improved the accuracy and reliability of nodal staging. Furthermore, the elective nodal areas are located close to the parotid glands, the submandibular glands, and the swallowing muscles. To spare toxicity of these regions, irradiation of a smaller, more selected volume of the elective nodes is key. Several researchers consider the current bilateral elective neck irradiation strategies as overtreatment and show growing interest in unilateral nodal irradiation in selected patients, which should be only conducted in clinical trials (59, 60).

### 5.4 Single and/or total radiation dose

Even if a total-dose escalation did not lead to the hoped-for benefit in locally advanced laryngeal and hypopharyngeal cancers (61), escalation in the single dose, named moderate hypofractionation with doses of 2.2–2.5 Gy, showed a benefit in therapy outcome (62); also, six fractions a week instead of five showed a clear benefit but increased toxicity (63).

### 5.5 Radiotherapy in combination with immunotherapy

Until today, all phase 3 trials investigating the combination of immunotherapy with radiochemotherapy in the primary setting have yielded negative results. Radiation intervenes in preclinical models (*in vitro* in cell models as well as in animal models) with the same signaling pathways and mechanisms that are targeted with immunotherapy approaches (e.g., PD1-PD-L1 axis, and TGFbeta). In addition, the infiltration of immune cells in the tumor microenvironment is changed as well as the functionality of the immune cells. Therefore, radiation can have both positive and negative effects on the anti-tumor immune response (64). The immunological effect of radiation depends on patient and tumor factors, as well as on radiation oncological factors (e.g., radiation dose, fractionation, irradiated volume, and radiation modality). Different radiation doses have different pro- and anti-immunogenic effects, so an “ideal” radiation dose in the sense of a one-size-fits-all cannot be determined (65). Preclinical data indicate that irradiation of the lymphatic drainage pathways significantly inhibits the anti-tumor immune response (66). Irradiation of lymph node vessels is considered the reason for unsatisfactory results of combined radiotherapy and immunotherapy in phase III studies. However, the decision to spare or irradiate lymph nodes electively should be evidence-based and balance the nodal risk against any presumed immunologic or functional benefit (67, 68).

Further points such as selective nodal irradiation, omission of the resected neck, and artificial intelligence (radiomics) are finding their way as therapy options at a rapid pace, although not yet in the routine. In summary, for optimal therapy, we have to coordinate all options with each other optimally (therapy team).

## 6 New options for neoadjuvant treatment including immune checkpoint inhibitors

Currently, we learn from several ongoing and finalized NCT trials with the integration of immune checkpoint inhibitors (NICT) about the high potential of this new therapeutic option. Uppaluri et al. presented the first preliminary data of two cycles of neoadjuvant pembrolizumab monotherapy before surgery in 36 patients and concluded that among patients with locally advanced, human papillomavirus (HPV)-unrelated HNSCC, pembrolizumab was safe, and no pathological response was observed in 44% of patients with 0% pathological complete responses. The 1-year relapse rate in patients with high-risk pathology was lower than in comparable studies using classical chemotherapy. Surgery was technically feasible, and no intraoperative and wound healing problem was observed regarding assumed higher tissue blood perfusion due to pro-inflammatory treatment (69). Following these preliminary data, MSD initiated the KEYNOTE-689 trial, a phase 3 study of adjuvant and neoadjuvant pembrolizumab combined with standard of care (SOC) in 600 patients with resectable, locally advanced HNSCC. The recent press release announced that the event-free survival (EFS) was significantly improved with the addition of pembrolizumab.

Zuur and her team from Amsterdam presented the first data of the IMCISION trial, a non-randomized phase Ib/IIa trial. A total of 32 HNSCC patients were treated with two doses (in weeks 1 and 3) of immune checkpoint blockade using nivolumab (NIVO MONO, n = 6, phase Ib arm A) or nivolumab plus a single dose of ipilimumab (COMBO, n = 26, 6 in phase Ib arm B, and 20 in phase IIa) prior to surgery. Pathological response, defined as the percent change in primary tumor viable tumor cell percentage from baseline biopsy to on-treatment resection, was evaluated in 17/20 phase IIa patients and 29/32 total trial patients (6/6 NIVO MONO and 23/26 COMBO). We observed a major pathological response (MPR; 90%−100% response) in 35% of patients after COMBO ICB, both in phase IIa (6/17) and in the whole trial (8/23), meeting the phase IIa primary endpoint threshold of 10%. NIVO MONO’s MPR rate was 17% (1/6). None of the MPR patients developed recurrent HNSCC during 24.0 months of median postsurgical follow-up. As a side note, this is the only trial showing any advantage for combination therapy of PD-1+CTLA-4 blockade compared to anti-PD1 mono in head and neck cancer per se (70).

Another encouraging trial was the CheckRad-CD8 trial from Hecht et al., Germany (71). A total of 56 patients received a single cycle of cisplatin 30 mg/m^2^ on days 1–3 and docetaxel 75 mg/m^2^ on day 1 combined with durvalumab 1,500 mg fixed dose on day 5 and tremelimumab 75 mg fixed dose on day 5. Patients with pathological complete response (pCR) in the rebiopsy after induction treatment or at least 20% increase of intratumoral CD8+ cell density in the rebiopsy compared with baseline entered radioimmunotherapy with concomitant durvalumab/tremelimumab. The objective of this interim analysis was to analyze the safety and efficacy of the chemoimmunotherapy-induction treatment before radioimmunotherapy. After induction treatment, 27 patients (48%) had a pCR in the rebiopsy, and a further 25 patients (45%) had a relevant increase of intratumoral CD8+ cells (median increase by a factor of 3.0). On multivariable analysis, intratumoral CD8+ cell density predicted pCR independently. Following this observation, to determine whether a single dose of double immune checkpoint blockade [induction chemoimmunotherapy (NICT) CheckRad-CD8 protocol] adds benefit to induction single-cycle platinum doublet (induction chemotherapy NCT) in locally advanced HNSCC, patients treated with immune checkpoint inhibitor therapy (ICIT) within the CheckRad-CD8 trial were compared with a retrospective cohort receiving the same chemotherapy without immunotherapy. The endpoint of this analysis was the complete response (CR) rate. A total of 53 patients were treated with ICIT, and 104 patients were treated with NCT only. Remarkably, CR rates were 60.3% for ICIT and 40.3% for IC (p = 0.018) (72).

Other highly stimulating small, uncontrolled single institution trials with a focus on neoadjuvant immunochemotherapy for locally advanced resectable oral HNSCC with new data come from China. The ILLUMINATE trial is a prospective trial of NICT with toripalimab (PD-1 inhibitor) and albumin paclitaxel/cisplatin (TTP) was conducted in 20 patients with clinical stage III and IVA oral squamous cell carcinoma (OSCC). The MPR was 60%, including a 30% pathological complete response with no obstruction of subsequent surgery. During the median 23-month follow-up, the disease-free survival was 90%, and the overall survival was 95% (73). Another phase I trial was published, treating 20 patients with locally advanced resectable oral HNSCC with three cycles of camrelizumab (anti-PD-1) and apatinib (VEGFR2 inhibitor) before surgery. Neoadjuvant treatment was well-tolerated, and the MPR rate was 40% (8/20). All five patients with a combined positive score (CPS) >10 achieved MPR. *Post-hoc* analysis showed 18-month locoregional recurrence and survival rates of 10.5% (95% CI: 0%–24.3%) and 95% (95% CI: 85.4%–100.0%), respectively (74). Huang et al. published a phase 1b trial with neoadjuvant toripalimab combined with gemcitabine and cisplatin in 23 patients with resectable locally advanced HNSCC (NeoTGP01) (75). The overall response rate (ORR) reached 45%. Eighteen patients underwent successful surgical resection. The R0 resection rate was 100%. The pathological response rates were 16.7% (pCR), 27.8% (MPR; two of five near-pCR). Finally, Zhang et al. presented data from a single-center, single-arm, phase 2 trial (76). A total of 30 patients with resectable stage III–IVB HNSCC received chemotherapy [albumin-bound paclitaxel 260 mg/m^2^ (or docetaxel 75 mg/m^2^) plus cisplatin 75 mg/m^2^] and camrelizumab 200 mg (PD-1 inhibitor) on day 1 of each 21-day cycle for three cycles, followed by surgery and adjuvant radiotherapy. The pCR rate was 37.0%, and the MPR was 74.1% (95% CI, 53.7%–88.9%). The median follow-up duration was 16.1 months (range, 8.3–28.5), and the disease-free survival rate at 12 months was 95.8% (95% CI, 73.9%–99.4%). All data presented here are highly exciting and generate relevant hypotheses for future controlled multicenter phase II and III studies to establish NICT before surgery in advanced HNSCC.

**Figure 4:**
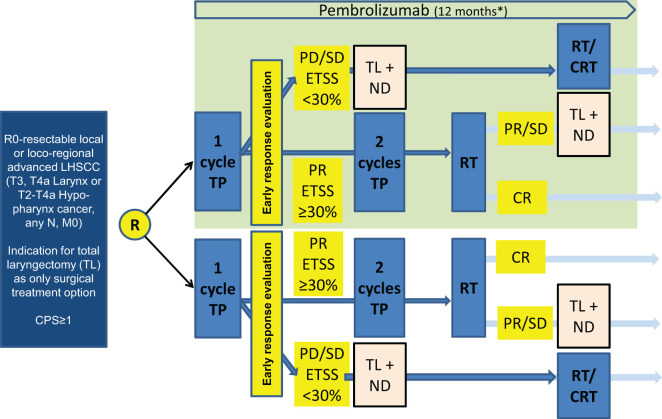
ELOS flowchart. Induction chemotherapy with docetaxel and cisplatin followed by radiation compared to additional PD-1 inhibition in CPS ≥1 advanced laryngeal/hypopharyngeal cancer suitable for laryngectomy selected after early response evaluation (78). CPS, combined positive score; R, randomization; TP, induction chemotherapy utilizing docetaxel (T) and cisplatin (P). Early response evaluation according to DeLOS-II criteria: PR, partial response ≥30% endoscopic tumor surface shrinkage (ETSS) after one cycle; PD/SD, progressing disease or insufficient response <30% ETSS; TL, total laryngectomy; ND, neck dissection; RT, radiotherapy; CRT, concomitant cisplatin-based chemoradiotherapy; endoscopic evaluation. Medication and radiation protocol according to the DeLOS-II LOP trial with additional pembrolizumab (over 6 months) in the experimental arm (light green). * meaning is: Pembrolizumab will be applicated for one year maintenance.

In regard to larynx organ preservation, the traditional domain of NCT, Ferrarotto et al. presented at American Society of Clinical Oncology (ASCO) 2023 (77) the first very interesting data of immuno-chemotherapy [pembrolizumab (P), cisplatin (C), and docetaxel (D)] as a single treatment modality for larynx preservation (ICoLP) in 23 patients. Disease control rate was 100% with 74% (17/23) being objective responses and 52% CR; pathological CR rate was 77.3% (17/22; one patient was on-treatment). Six of 17 (35%) patients with pCR developed recurrence, mostly (4/6) within 4 months of pCR, and were salvaged with laryngectomy. In Germany, the interdisciplinary working group for head and neck cancer (IAG-KHT) started this year the ELOS trial, a prospective, randomized, open-label, controlled, two-armed parallel group, phase II multicenter trial in local advanced stage III, IVA/B head and neck squamous cell carcinoma of the larynx or hypopharynx (LHNSCC) with PD-L1 expression within tumor tissue biopsy, calculated as CPS ≥ 1 curable by total laryngectomy. Induction chemotherapy (IC) with docetaxel and cisplatin (TP) followed by radiation will be compared to additional PD-1 inhibition. Patients will be selected after short induction early response evaluation after the first cycle IC (IC-1) aiming at larynx organ preservation by an additional two cycles of IC followed by radiotherapy (69.6 Gy) for responders achieving endoscopic estimated tumor surface shrinkage (ETSS) ≥30%. Non-responders (ETSS < 30% or progressing disease) will receive total laryngectomy and selective neck dissection followed by postoperative radiation or chemoradiation according to the recommendation of the clinic’s multidisciplinary tumor board. Patients randomized into the intervention arm starting day 1 will receive 200 mg pembrolizumab in a 3-week cycle for 17 cycles (12 months). Treatment with pembrolizumab will continue in the experimental arm regardless of ETSS status after IC-1 in both responders and laryngectomized non-responders, independent from the subsequent decision on adjuvant therapy after TL (78, **Figure 4**). The study is based on the encouraging experience of the abovementioned DeLOS-II trial (25).

## 7 Conclusion

It is likely that improved patient selection, refinements in radiotherapy technique, and drug combinations will provide different outcomes from those obtained in RTOG 91-11 patients treated more than 20 years ago. Good decision-making requires familiarity with decision-relevant factors and recognition of the values relevant to weighing the pros and cons of the alternatives, i.e., in advanced LHSCC balancing functional larynx preservation and oncologic safety. In the last few years, NCT in combination with immune checkpoint inhibitors (NICT) as part of perioperative transoral surgical treatment concepts of advanced HNSCC is gaining interest again due to increasing response rates and functional and overall survival outcomes. NICT has been shown to be effective in non-controlled, small but many different, trials and opens a new door for new surgical concepts. Today, NCT/NICT in combination with transoral surgery or radiation is not a standard treatment. However, the topic is highly relevant and should stimulate worldwide the surgical community to perform NCT/NICT clinical trials focusing on LOP, gaining precision in better selection of responders, improving the rate of long-term larynx preservation, and limiting toxicity. This seems to be the challenge for the improvement of concepts in head and neck surgery with a focus on better survival and functional organ preservation of our patients for the next decade.
